# Case Report: Placental Maternal Vascular Malperfusion Affecting Late Fetal Development and Multiorgan Infection Caused by SARS-CoV-2 in Patient With PAI-1 4G/5G Polymorphism

**DOI:** 10.3389/fmed.2021.624166

**Published:** 2021-02-17

**Authors:** Behling JAK, Gabriele Zanirati, Felipe V. F. Rodrigues, Matheus Grahl, Felipe Krimberg, Giulia Pinzetta, Larissa Borém, Daniela Savi, Denise Cantarelli Machado, Jaderson Costa Da Costa, Daniel Rodrigo Marinowic

**Affiliations:** ^1^Brain Institute of Rio Grande do Sul (BraIns), Pontifical Catholic University of Rio Grande do Sul, Porto Alegre, Brazil; ^2^Graduate Program in Medicine and Health Sciences, Medical School, Pontifical Catholic University of Rio Grande do Sul, Porto Alegre, Brazil; ^3^Graduate Program in Medicine, Pediatrics and Child Health, Medical School, Pontifical Catholic University of Rio Grande do Sul, Porto Alegre, Brazil; ^4^Graduate Program in Health Woman, Medical School, Federal University of Minas Gerais, Horizonte, Brazil; ^5^Medical Pathologist, Virchow Laboratory, Belo Horizonte, Brazil; ^6^Graduate Program in Biomedical Gerontology, Medical School, Pontifical Catholic University of Rio Grande do Sul, Porto Alegre, Brazil

**Keywords:** SARS-CoV-2, placenta infection, vascular malperfusion, multi-organ infection, vertical transmission

## Abstract

**Background:** Pregnant women are susceptible to the novel coronavirus (SARS-CoV-2), and the consequences for the fetus are still uncertain. Here, we present a case of a pregnant woman with subclinical hypothyroidism and a plasminogen activator inhibitor type 1 (PAI-1) 4G/5G polymorphism who was infected with SARS-CoV-2 at the end of the third trimester of pregnancy, with unexpected evolution of death of the newborn 4 days postpartum.

**Methods:** Nested PCR was performed to detect the virus, followed by ssDNA sequencing.

**Results:** Transplacental transmission of SARS-CoV-2 can cause placental inflammation, ischemia, and neonatal viremia, with complications such as preterm labor and damage to the placental barrier in patients with PAI-1 4G/5G polymorphism.

**Conclusion:** We showed a newborn with several damages potentially caused due to the PAI-1 polymorphisms carried by the mother infected with SARS-CoV-2 during pregnancy.

## Introduction

In December 2019, several cases of pneumonia emerged in the city of Wuhan, China. It was found that it was due to a new betacoronavirus named severe acute respiratory syndrome coronavirus 2 (SARS-CoV-2). The disease spread rapidly, and subsequently, SARS-CoV-2-infected patients were identified in other countries around the world. In March 2020, the World Health Organization (WHO) declared the disease a pandemic (1, 2).

Infection with the SARS-CoV-2 virus causes Coronavirus Disease 2019 (COVID-19), whose main symptoms are fever, fatigue, and cough, which may progress to dyspnea or, in more severe cases, severe acute respiratory system (SARS) (3). An older age and comorbidities, such as diabetes, respiratory disease, hypertension, severe heart disease, or immunosuppression, are considered risk factors for worse outcomes of coronavirus infection.

There is some evidence indicating that pregnant women are also susceptible to this new coronavirus; however, there are only limited data regarding the effects of SARS-CoV-2 infection during pregnancy. There is evidence that pregnant women are more susceptible to respiratory pathogens, including SARS and Middle East Respiratory Syndrome (MERS), which caused severe complications during pregnancy (4–6). Recently, a few reports have suggested that pregnant women are also susceptible to SARS-CoV-2 (7, 8). Additionally, a report showed that the transplacental transmission of SARS-CoV-2 is possible (9). However, the consequences for the fetus are still uncertain.

Here, we present a case of a pregnant woman with subclinical hypothyroidism and the presence of a plasminogen activator inhibitor type 1 (PAI-1) 4G/5G polymorphism condition who was infected with SARS-CoV-2 at the end of the third trimester of pregnancy in southeast Brazil, and we demonstrated vertical transmission of SARS-CoV-2. The present study discusses the details of the fetal multiorgan tissue virological and pathological investigations.

## Case Report

A 36-year-old pregnant woman, in her first pregnancy, presented for prenatal care at a high-risk pregnancy service due to subclinical hypothyroidism and the presence of a PAI-1 4G/5G polymorphism. The prevalence of the PAI-1 polymorphism (4G/5G) is very high in the general population, ~50%, and there is no evidence that it influences the outcomes or progress during pregnancy. During her prenatal care, there were no maternal or fetal complications.

An ultrasound scan at 37 weeks of gestation to assess growth and fetal well-being showed good progression. The estimated fetal weight was 2,920 g (40th centile), amniotic fluid index 14.8 cm, normal maternal and fetal Doppler [mean uterine artery PI = 0.51, umbilical artery with positive diastolic flow and pulsatility index (PI) = 1.05, middle cerebral artery (MCA) PI = 1.19, a normal cerebroplacental ratio (CPR) = 1.13]. The biophysical profile was also normal (8/8).

At the 39th week of gestation, she presented with spontaneous rupture of the membranes before labor with meconium amniotic fluid, and she started contractions of the active phase afterward. A physical exam revealed a normal cardiotocography of category 1, a reassuring pattern, and no abnormalities, normal uterine tone, satisfactory uterine dynamics, a 6-cm-dilated cervix, and a cephalic fetus. She received epidural analgesia on request.

After 5 h of adequate labor progression, she entered the expulsive period, and operative vaginal delivery (fetal extraction with Simpson forceps) was necessary due to a non-reassuring fetal status. A newborn male, hypotonic, weighing 2,600 g, was extracted. His Apgar scores were 1, 1, and 4 in the 1st, 5th, and 10th min, respectively. He was referred immediately to the neonatal intensive care unit. Death occurred after 4 days.

There was an extensive placental lesion that severely compromised the fetal perfusion. Considering the unfavorable outcome of the case, and understanding that there is no evidence in the literature that the PAI 1 4G/5G polymorphism could result in this type of ischemia, taking into account the context of the current pandemic and the recent evidence correlating SARS-COV-2 infection to placental injury, at 10 days after the delivery, the patient's serology was evaluated even without having symptoms. Positive IgG, (10.9 UA/ml) and negative IgM (0.2 UA/ml), was detected by the immunofluorescence method, so it was decided to test the fetus and placenta as well.

## Ethical Aspects

This study was approved by the Research Ethics Committee of Pontifical Catholic University of Rio Grande do Sul (PUCRS) with approval number 3.977.510. The participant provided written informed consent before inclusion in this study.

## Methods

### RNA Extraction and Reverse Transcription for First-Strand DNA Synthesis

RNA was extracted from paraffin-embedded samples of the placenta, lung, liver, heart, kidney, and brain of the fetus using a ReliaPrep™ FFPE Total RNA Miniprep System kit (PROMEGA) according to the manufacturer's recommendations. The RNA control was extracted from nasopharyngeal and oropharyngeal (throat) specimens collected by a healthcare professional following the Centers for Disease Control and Prevention (CDC) instruction guidelines using an SV-Total RNA kit (PROMEGA).

Reverse-transcriptase first-strand DNA synthesis was performed by the 3′ primer technique using M-MLV reverse transcriptase (Thermo Fisher Scientific) with two distinct reverse primers (hCOVassay1 R: 5′-AGCAGCATCACCGCCATTG-3′ and hCOVassay2 R: 5′-CCGCCATTGCCAGCCATTC-3′). After the transcription reaction, the product was quantified in a NanoDrop fluorometer (Thermo Fisher Scientific).

### Molecular Analysis Using qRT-PCR

Real-time PCR was performed using StepOne Plus (Thermo Fisher Scientific) equipment. The samples were amplified from the initial amount of over 200 ng of single-stranded DNA (ssDNA) for each sample using the PowerUp SYBR Green Master Mix kit (Thermo Fisher Scientific). The primer sequences used were hCOVassay1–F 5′-GCCTCTTCTCGTTCCTCATCAC-3′/R 5′-AGCAGCATCACCGCCATTG-3′ and hCOVassay2–F 5′-AGCCTCTTCTCGTTCCTCATCAC-3′/R 5′-CCGCCATTGCCAGCCATTC-3′.

### *Nested* PCR Technique

After the first reaction of RT-PCR, a new amplification was performed using the same primer set as the first PCR. For this new reaction (*nested* PCR), the PCR product generated in the initial amplification was used as a template. The thermal cycles and the optimized times were the same as those used for the first PCR amplification.

### ssDNA Sequencing

Sequencing of the samples was performed by ACTGene Análises Moleculares Ltd. (Center for Biotechnology, UFRGS, Porto Alegre, RS, Brazil) using an AB 3500 Genetic Analyzer (Thermo Fisher Scientific). Sequencing data were collected using Data Collection 3 software (Thermo Fisher Scientific), and the resulting Data Collection files were converted into FASTA files using standard parameters. Using Clustal Omega software, the FASTA files were aligned along with the complete Sars-CoV-2 genome sequences as follows: China (MT079844.1), Italy (MT890669.1), USA (MT642254.1), Russia (MT890462.1), and Brazil (MT827074.1).

## Results

### Autopsy and Organ Findings

Necropsy showed alterations compatible with septic shock, pulmonary congestion, hypoxia, acute tubular necrosis in the kidneys, and hemorrhagic alterations in the adrenals. Moreover, it demonstrated acute heart failure with liver failure and signs of fetal distress.

The placenta and umbilical cord weighed 416.0 g and measured 42.0 × 12.0 × 4.0 cm. The placenta shape was discoid, with a firm reddish-colored maternal face, spongy in appearance, and adherent clots. It was also possible to observe diffuse whitish areas. The fetal face of the placenta was smooth and opaque and showed a membrane with evident vessels. On the histological examination, the placenta showed extensive areas of deposits of intervillous and subchorionic fibrin, present in the entire length of the examined fragments. There were recent placental infarctions, villus agglutination, and narrowing of the intervillar spaces, an increase in the number of syncytial nodes. Presence of fetal thrombotic vasculopathy in the large vessels in the region where the umbilical cord is implanted and in the septal vessels. Intense congestion of the vessels and recent hemorrhage of the perivascular jelly in the umbilical cord, with streaks of fibrin amidst the red blood cells. This suggests uteroplacental malfusion and fetal vascular malfusion, leading to fetal hypoxia.

The left fetal lung showed intense and extensive acute bronchopneumonia, with numerous neutrophils and pyocytes filling the alveoli, along with abundant amniotic fluid, fibrin deposits, and growth of gram-positive bacterial colonies in blood culture. The interlobular septa showed intense congestion and marked recent hemorrhage, forming a trabecular aspect on macroscopic examination. The right lung showed intense capillary and vascular congestion, with amniotic fluid and meconium in the alveoli. In addition, hyaline membranes covered the walls of several alveoli and bronchioles. Several megakaryocytes were also observed in the capillary of the alveolar septa.

**Figure 1:**
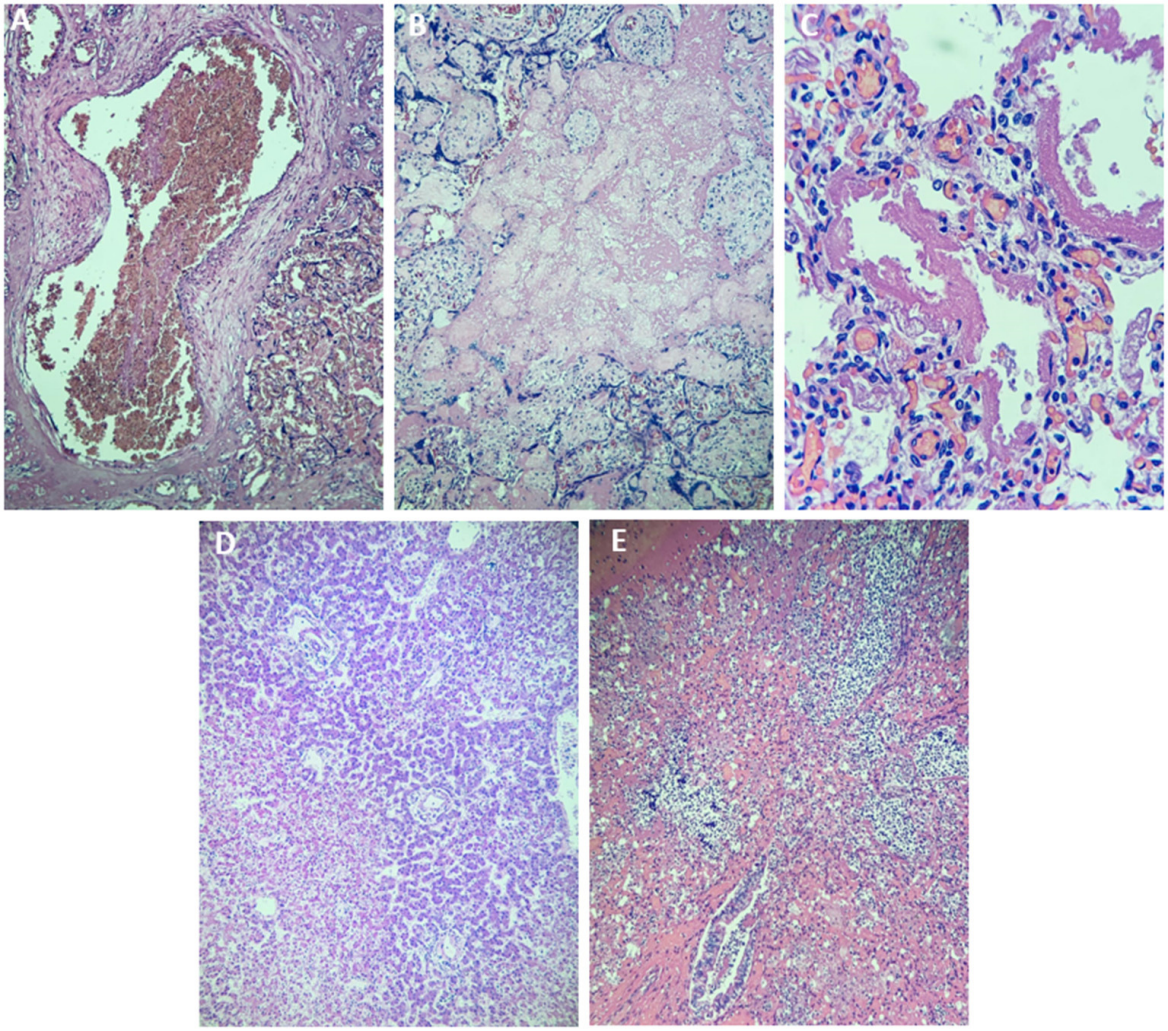
Histopathology of different tissues of the newborn using hematoxylin and eosin staining. **(A)** Thrombus in a placental blood vessel in the region of the insertion of the umbilical cord (×40). **(B)** Thrombosis of the placental intervening space (×40). **(C)** Lung with hyaline membranes (×400). **(D)** Hepatocellular necrosis in the hepatic centrilobular region (×40) **(E)**. Characteristic image of acute bronchopneumonia (×100).

The heart and brain did not demonstrate significant changes, with normal neural and glial cellularity in the brain and cerebellum. The liver showed lobulated hepatocellular parenchyma with intense extracellular (canalicular) and more discrete intracellular cholestasis, with necrosis of the hepatocytes in the lobular center perivein, where recent hemorrhage and congestion were also observed. The right adrenal had 5.0 mm necrosis of the cortex, associated with recent peripheral and medullary hemorrhage. The left adrenal had intense congestion and recent hemorrhage in the cortex. The kidneys presented a mature appearance, showing intense congestion and foci from recent hemorrhage, in addition to hyaline cylinders in the tubules and diffuse acute tubular necrosis. The spleen, pancreas, and gallbladder did not show any noticeable alterations (Figure 1).

### Virology Investigation

**Figure 2:**
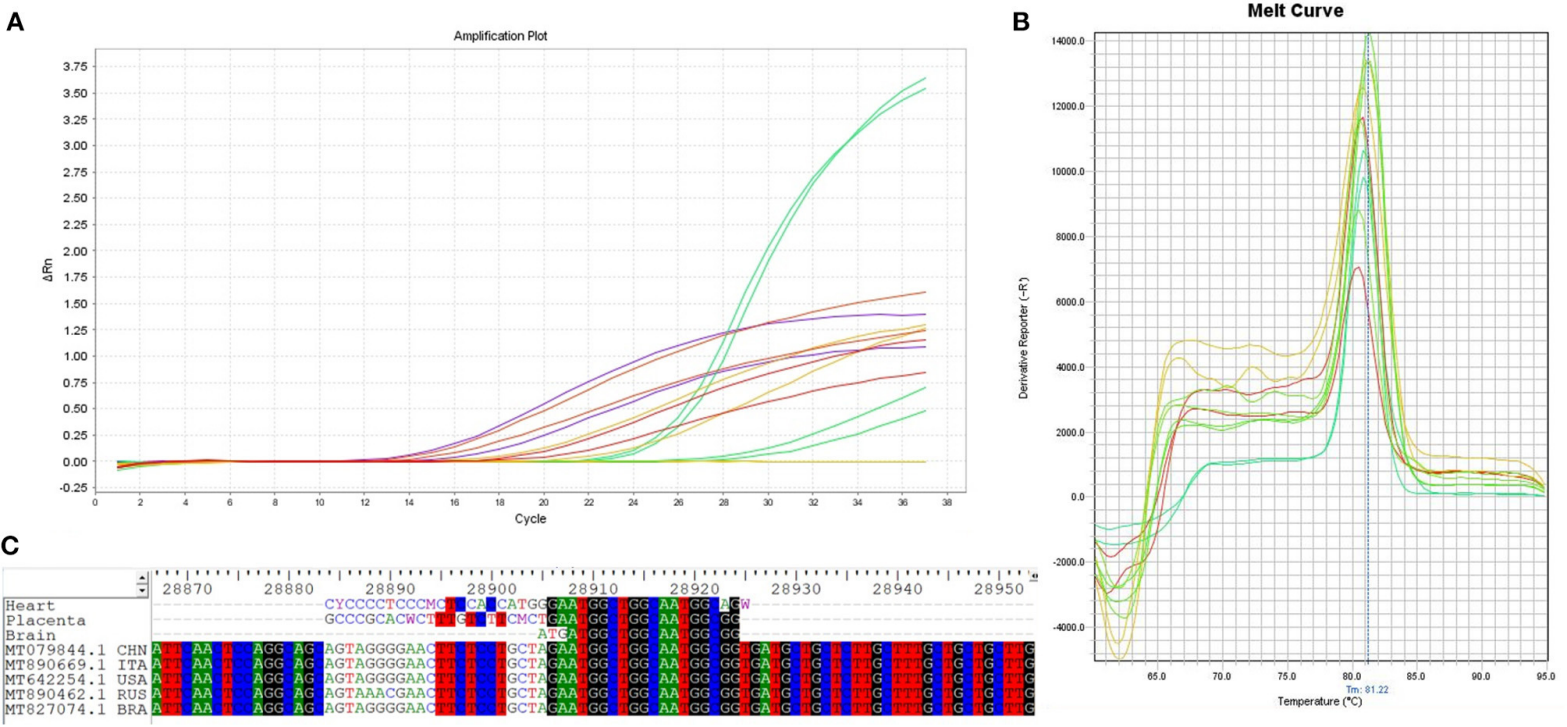
Real-time PCR amplification, melt curves, and ssDNA sequencing of SARS-CoV-2 from different tissues of the newborn. **(A)** Amplification curve of SARS-CoV-2 in samples from the placenta, lung, heart, liver, and brain. **(B)** The melt curves showed a similar pattern for all SARS-CoV-2-positive amplifications detected in all tissues analyzed. **(C)** ssDNA sequencing of generated amplicons. The results obtained by sequencing the amplicons showed high homology with SARS-CoV-2 sequences. The homology analysis was performed using the entire genome sequences of copies of the SARS-CoV-2 virus from Russia (MT890462.1 RUS), USA (MT642254.1 USA), Italy (MT890669.1 ITA), China (MT079844.1 CHN), and Brazil (MT827074.1 BRA).

It was possible to detect the presence of SARS-CoV-2 through a *nested* RT-PCR assay in the placenta, liver, heart, lung, and brain samples. Simple RT-PCR assays did not identify the virus in any of the tissues analyzed. The amplification and melt curves are shown in Figure 2. The sequencing of the generated amplicons showed a high sequence identity for different strains of SARS-CoV-2. The placenta showed a sequence identity of 100% with a query cover of 46%, the brain demonstrated an identity of 100% with a query cover of 45%, and in the heart, 100% identity with a query cover of 40%. It was not possible to analyze the sequences in the other organs due to the low quantity of material.

## Discussion

In this case report presented, we demonstrate the transplacental transmission of SARS-CoV-2 from a pregnant woman with PAI-1 polymorphism (4G/5G) who evolved to unexpected fetal death. Despite extensive support, there was massive placental injury and fetal death occurred 4 days after birth, which we believe has a correlation with a probable COVID-19 infection at the end of pregnancy, which was diagnosed postpartum in view of the unexpected outcome.

Thrombophilia is a hereditary or acquired condition that can cause an increased risk of venous thromboembolism (10). There is a common polymorphism in the promoter region of the PAI-1 gene that can cause thrombophilia. PAI-1 is a protein secreted in response to inflammatory reactions that participates in hemostasis through its regulatory activity on the fibrinolysis process, inhibiting plasminogen cleavage and thus clot dissolution. The 4G allele is associated with increased gene expression, resulting in higher circulating PAI-1 concentrations, while the presence of the 5G allele results in lower PAI-1 levels.

Most obstetric societies worldwide recommend not performing routine PAI-1 polymorphism testing (10, 11). Screening for PAI-1 should not be performed since there is no evidence that it could have any influence on adverse outcomes during pregnancy or affect patient management, since approximately 50% of the general population presents with this polymorphism (10). Thus, including PAI-1 in routine screening could generate anxiety and adverse effects of unnecessary conduct (11).

However, the current discussion is about whether COVID infection can influence hereditary thrombophilias, as well as acquired thrombophilias, or even potentiate polymorphisms that under normal conditions would not present significant changes.

The ideal management of pregnant women with COVID-19 presents several challenges, although it was originally thought that pregnant women with COVID-19 were not likely to develop severe morbidity or progress to death, recent reports suggest that a subset may develop multiple organ failure and even die. Given that healthy pregnant women have evidence of increased thrombin generation and a prothrombotic state, as well as increased intravascular inflammation that is exaggerated against the background of infection, such patients may have an increased risk of thrombosis when affected by COVID-19.

The International Society of Thrombosis and Hemostasis generated a simple algorithm for the management of COVID-19 coagulopathy (12). It was recommended that low molecular weight heparin be considered in all of these patients. This set of evidence should be considered by obstetricians who care for pregnant women with COVID-19. A coagulation profile to detect the presence of subclinical disseminated intravascular coagulation and the use of low molecular weight heparin for the prevention of thromboembolic disorders should be considered and discussed by physicians and patients. In this context, care should also be increased in the setting of smoking and contraception or replacement of ovarian hormones (10).

Although not conclusive, we demonstrated a possible correlation between potentiation of hereditary thrombophilia by COVID with an unfavorable progression of the case report presented. The patient's polymorphism, as stated earlier, is not a formal indication of prophylaxis for thromboembolism, the pregnant woman already arrived in prenatal care on medication, insecure in suspending, chose to keep use, even after the associated risks were explained and not have indication to keep medication during pregnancy (12, 13).

Gestational success depends on adequate uteroplacental circulation. Abnormalities in this vascular network are related to several gestational pathologies, including miscarriages, fetal death, restricted intrauterine growth, preeclampsia, and premature placental detachment (14, 15).

Interestingly, these results, associated with the clinical history, suggest an alteration of coagulation, hereditary or acquired (thrombophilia and viruses), or even an association of both. This suggests there may be complications among patients with thrombophilia and a confirmed SARS-CoV-2 infection.

It should be emphasized that both the placenta and organs of the newborn had laboratory confirmation of SARS-CoV-2 infection by reliable methods, and all histopathological tests were performed by the same experienced pathologist, blinded to the maternal results of the SARS-CoV-2. One limitation of our case series is that we did not collect amniotic fluid, and the blood analysis of the fetus showed negative SARS-CoV-2 results. However, because it is a pregnancy that progressed to fetal death, we found evidence of the presence of SARS-CoV-2 in several organs analyzed *post mortem*, as well as in the placenta, thus proving transplacental transmission. We also do not know whether fetal viremia and the presence of SARS-CoV-2 in the tissues may have influenced in some way the response to the treatments performed without success.

This case of fetal death in a woman with the presence of the PAI-1 4G/5G mutation and evidence of SARS-CoV-2 infection at the end of pregnancy strongly suggest their combination can cause complications related during pregnancy. The intense placental inflammatory reaction, including villitis and intervillositis, as well as the association with fetal thrombotic vasculopathy, raises the possibility of a direct effect of SARS-CoV-2 on the placenta (13, 14). The identification of SARS-CoV-2 in placenta samples corroborates this hypothesis, in addition to suggesting its exacerbation in patients with preexisting comorbidities such as thrombophilia, since the autopsy showed signs of fetal infection and coagulation alteration (16, 17).

Additionally, other studies have shown that transplacental transmission is indeed possible in the last weeks of pregnancy (18). Overall, there is limited evidence of vertical transmission or significant mortality among pregnant women with COVID-19. However, several adverse perinatal outcomes have been reported, including an increased risk of miscarriage, premature rupture of the membranes, premature and stillbirth, and preeclampsia (16). In this report, we highlight the lack of weight gain predicted during the critical period of the 37th to 40th weeks, where fetal growth is an extremely important factor for the outcome and good fetal progression, showing complications of restricted intrauterine growth due to alterations in the cord and placental perfusion, leading to hypoxia and unfavorable evolution to fetal death. This fact is corroborated by the 37-week ultrasound that presented good outcomes with an estimated fetal weight of 2,920 g (40th percentile), the birth weight counterpart of 2,600 g at 39 weeks (near the 3rd percentile). This fact reinforces the hypothesis that placental injury occurred during the last weeks of pregnancy, leading to a placental flow deficiency that culminated in chronic fetal hypoxia and failure of the expected lack of weight gain.

During pregnancy, the placenta acts as a physical and immunological barrier against hematogenous transmission of viruses from the mother to the fetus. Nevertheless, very little is known about the specific mechanisms by which the placenta protects the developing fetus from viral infections or about the strategies used by viruses to circumvent and/or weaken the placental barrier (16, 17).

However, the exact mechanisms of intrauterine transmission of SARS-CoV-2 are not well defined. One of them includes angiotensin-converter enzyme 2, as a possible surface receptor of SARS-CoV-2-sensitive cells, which is expressed in the human placenta. This could explain the placental infection with the virus. Another possible explanation for intrauterine infection by SARS-CoV-2 is the damage to the placental barrier caused by severe maternal hypoxemia in women with COVID-19, which in the case of thrombophilia would justify vascular involvement and coagulation (15).

Because it is a new disease and especially when talking about COVID-19 in pregnancy, it is important to report all adverse outcomes potentially associated with this disease. These data are important to inform patients and health professionals and to develop appropriate management protocols for pregnant women with COVID-19. As the global epidemic continues to expand, there will be additional information available on the effects of COVID-19 on pregnant women and their newborns (13). In the unfortunate event of mortality resulting from SARS-CoV-2 infection among pregnant or newborn women, pathological evaluation of the tissues together with molecular characterization of the virus would be useful in determining the pathogenesis of the disease, as is the case in all emerging infections (17, 18).

As the global epidemic continues to expand, there will be additional information available on the effects of COVID-19 on pregnant women and their newborns (13). In the unfortunate event of mortality resulting from SARS-CoV-2 infection among pregnant or newborn women, pathological evaluation of tissues together with molecular characterization of the virus would be useful in determining the pathogenesis of the disease, as is the case in many cases of emerging infections (17, 18).

In conclusion, we showed the possibility of transplacental transmission of SARS-CoV-2 infection during the last weeks of pregnancy. In addition, transplacental transmission can cause ischemia and malperfusion, with complications such as preterm labor and damage to the placental barrier in patients with the PAI-1 4G/5G polymorphisms. Further studies are needed to confirm our findings and help guide pregnancy management in women with COVID-19, especially in the last trimester of pregnancy in patients with risk factors such as thrombophilias.

## Data Availability Statement

The authors acknowledge that the data presented in this study must be deposited and made publicly available in an acceptable repository, prior to publication. Frontiers cannot accept a article that does not adhere to our open data policies.

## Ethics Statement

Written informed consent was obtained from the individual for the publication of any potentially identifiable images or data included in this article.

## Author Contributions

BJ designed the protocols and wrote the manuscript. GZ helped with RNA extraction and performed the PCR assays. FR participated in the PCR experiments. MG helped in the PCR assays. FK helped with RNA extraction. GP participated in the RNA extraction. LB assisted the clinical data of the pregnant and newborn. DS performed the histopathological analysis. DM aided in the design of all experiments and contributed the reagents. JD supported the design of all experiments and contributed the reagents. DM coordinated the research project. All authors carefully reviewed the manuscript.

## Conflict of Interest

The authors declare that the research was conducted in the absence of any commercial or financial relationships that could be construed as a potential conflict of interest.
